# A Computational Approach for the Prediction of p53 and BCL-2 Protein–Protein Interactions

**DOI:** 10.3390/ijms27010244

**Published:** 2025-12-25

**Authors:** Colette Creamer, Victoria Neely, Hisashi Harada

**Affiliations:** Philips Institute for Oral Health Research, Massey Comprehensive Cancer Center, Virginia Commonwealth University, Richmond, VA 23298, USA

**Keywords:** cancer, p53, BCL-2, computational biology, molecular docking

## Abstract

p53 has long been studied as a major regulator in cellular pathways, resulting in a plethora of information on the structure and function of this protein as a frequently mutated tumor suppressor. Recent studies have demonstrated how the p53 transcription activation domain (TAD) interacts with the BH3-binding pocket of BCL-2 to regulate cell survival/death. While the in vitro studies on p53 and BCL-2 have frequently used truncated and stabilized proteins of p53 to ensure crystallization, these mutated proteins are not naturally observed in cells. Thus, it becomes important to find a way in silico to simulate how a full-length monomer with the unaltered sequence of wild-type (WT) or missense mutant (MT) p53 interacts with BCL-2. Our objective is to provide a predictive insight into how p53 monomers might interact with BCL-2 through the combination of previously published algorithms. Using pre-established computational techniques in silico, the interactions between p53 variants and BCL-2 were compared to existing crystals to ensure the validity of the current method, and the affinities were predicted to explore the strength of these interactions. Here, we found that this protocol was able to replicate some of the amino acid interactions identified in the previous literature, as well as identify affinities between each WT/MT p53 and BCL-2. Most major MT p53 variants are predicted to directly interact with BCL-2, but have a decrease in affinity compared to WT p53, suggesting a potential increase in BCL-2 survival activity. Together, the method described here can potentially be useful as a predictive workflow to inform future studies in vitro and in vivo.

## 1. Introduction

p53 is a nexus of cellular processes, including DNA damage response, hypoxia, and apoptosis. As the “guardian of the genome”, this protein is essential in maintaining cellular behaviors. Upon mutation, as is common in cancerous cells, p53 loses its tumor suppressor activity and becomes oncogenic in certain cases [1]. In the context of cell survival/apoptosis, evidence indicates that wild-type (WT) p53 can colocalize at the mitochondria and directly interact with the BCL-2 family proteins to stimulate apoptosis [2,3,4]. In contrast, missense mutant (MT) p53 can lead to increased cellular survival through a variety of mechanisms [2,5]. Thus, the question arises whether the ability of WT p53 to interact with the BCL-2 family proteins in a pro-apoptotic manner is abrogated upon mutation of p53. In vitro studies involving p53 and the BCL-2 family proteins have used stabilized variants of p53 or its partial domains to demonstrate interactions with the BCL-2 family proteins [6,7]. Examples of these alterations include the use of an ‘SN15 peptide’ containing the p53 residues Ser15-Asn29, or a p53 peptide constructed using residues 39–57 [6,7]. Ha et al. also used a chimeric version of BCL-2 (hybridized with BCL-X_L_) to produce stable proteins [7]. However, due to the technical challenges of crystallizing sets of interacting proteins without modifying the proteins to maintain stability [8], the full structure of the p53-BCL-2 complex as it is most likely to appear in cells remains elusive.

Given that the structure of this complex is essential for identifying potential impacts of MT p53 on interactions with the BCL-2 family members, there is a need to see how a monomer with the unaltered sequence of WT or MT p53 interacts with the BCL-2 family proteins. While there are studies that show portions of WT p53 interacting with the BCL-2 family members in vitro [9,10], it remains a challenge to visualize interactions of intact, full-length proteins. This is due to substituting critical amino acids to help stabilize complexes for crystallization or to focus on the domains of the p53 protein that would otherwise be too challenging to crystallize [8]. Although both truncation and amino acid substitution methodologies have their benefits, a major drawback to both techniques is that they inherently produce results that do not account for steric interactions that would physiologically occur in the protein of interest. This led to the hypothesis that using computational methods to predict the interactions between p53 and BCL-2 family members could potentially reproduce some in vitro results and provide a clearer perspective into how unmodified monomers might interact in cells.

Our computational approach in silico involving p53’s interactions with the pro-survival protein, BCL-2, has led to the generation of a predictive procedure that produces a collection of data that can identify potential protein–protein interactions (PPIs) and their affinities. This would allow users to collect preliminary data that would support their in vitro experimental designs. Once the results with WT p53 were verified against those in prior literature, this new method was applied to common MT p53 to study how the changes in amino acids in MT p53 altered the interactions with BCL-2. The results from this method showed that major MT p53 are predicted to have a decrease in their binding affinity for direct interactions with the hydrophobic pocket of BCL-2, suggesting a potential increase in BCL-2’s pro-survival activity. These results also demonstrated the relative accuracy of the use of these computational resources, leading to a new workflow to generate predictive models for guiding future experiments.

## 2. Results

### 2.1. Structure Prediction of p53 Through ESMFold Yields Relatively Confident Models

**Figure 1:**
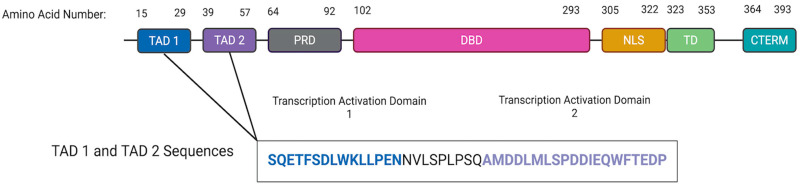
Overview of the Domains in p53. Transcription Activation Domain (TAD) 1 (blue), Transcription Activation Domain (TAD) 2 (purple), Proline Rich Domain (PRD) [18], DNA Binding Domain (DBD) [19], Nuclear Localization Signal (NLS), Tetramerization Domain (TD) [19], and the C-Terminus [19]. Created in BioRender. Creamer, C. (2025) https://BioRender.com/61gwui7.

**Figure 2:**
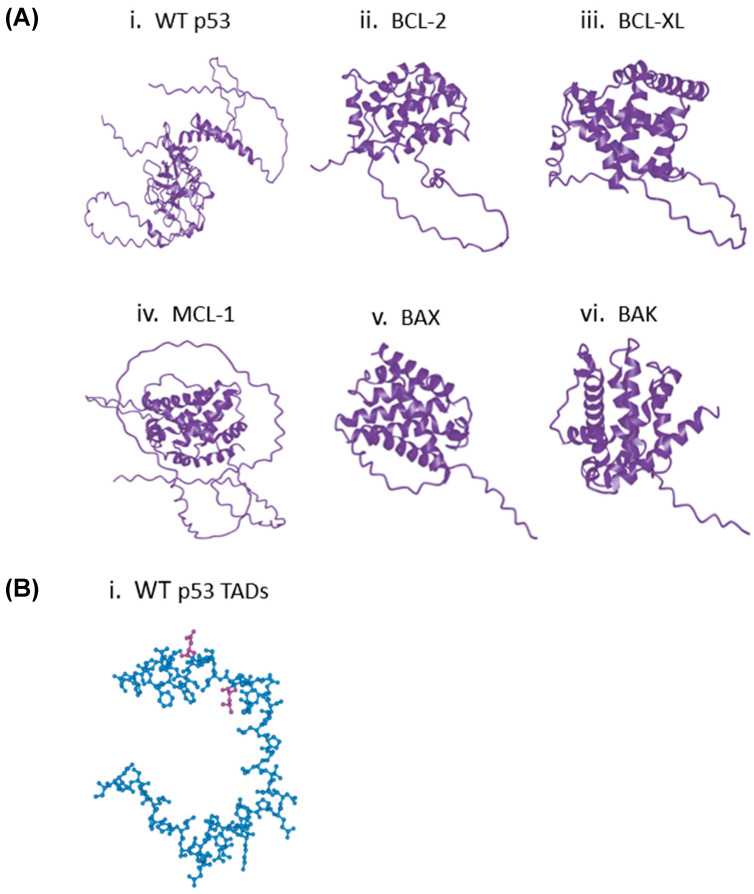
ESMFold Algorithm Produces Relatively Confident Protein Structure Models. (**A**) (i) WT p53, (ii) BCL-2, (iii) BCL-XL, (iv) MCL-1, (v) BAX, (vi) BAK. (**B**) (i) Predicted structure of WT p53 TADs. Purple residues are leucine (**top**) and aspartic acid (**bottom**).

To generate the structures necessary to predict the interactions between p53 (Figure 1) and the BCL-2 family proteins, two well-established protein structure prediction models, ESMFold [11] and AlphaFold [12], were used (Figure 2A and Figure S1). Both ESMFold and AlphaFold produced structures using the pLDDT score that had relatively high confidence in their predicted structures, apart from disordered regions (such as the p53 TADs (Figure 1 and Figure 2B and Figure S2) [13]. However, it is important to note that this method of structure reliability determination does not involve the superposition of structures. Thus, the reliability of our predicted WT p53 and BCL-2 structures was confirmed using the superposition tool FATCAT (https://fatcat.godziklab.org/fatcat/fatcat_homo.html (accessed on 21 December 2025)) (Riverside, CA, USA) [14,15,16,17]. The predicted structures generated from UNIPROT (Bethesda, MD, USA) sequences closely matched the Protein Data Bank (PDB) entries ‘5jsn’ for BCL-2 and ‘8r1f’ for p53 (Tables S1 and S2). Given this data, it was concluded that ESMFold and AlphaFold produced confident models of p53 and the BCL-2 family proteins that would be useful for further exploration in predicting protein–protein interactions.

### 2.2. Comparison of p53 TAD with BH3 Domains Indicates Similar Structure and Function in the Interactions with the BH3 Binding Pocket

**Figure 3:**
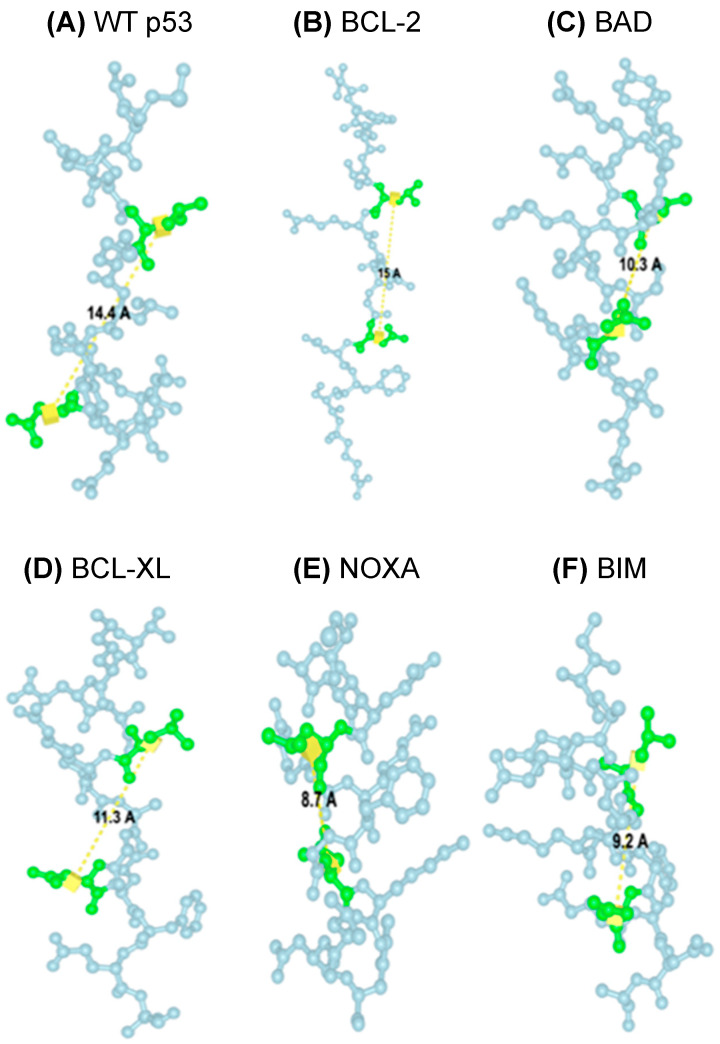
Identification and characterization of a putative BH3 motif in p53 TAD. Predicted lengths (yellow line) between L and D (green amino acids) in the BH3 motifs. (**A**) p53 (14.4 Å), (**B**) BCL-2 (15.0 Å), (**C**) BAD (10.3 Å), (**D**) BCL-XL (11.3 Å), (**E**) NOXA (8.7 Å), (**F**) BIM (9.2 Å).

Next, TAD1, TAD2, and the surrounding sequences in p53 (Figure 1) were characterized to see if there were any similarities to the BH3 motifs commonly seen in the BCL-2 family proteins. A potential similarity to the BH3 motif in amino acids 39–52 spanning both p53 TADs was found by aligning these amino acids with several known BH3 motifs (Figure 2B and Table 1) [7,17]. Critically, this portion of TAD has an LXXXxD motif (leucine followed by 3 of any amino acid, then a fourth small amino acid such as glycine, alanine, or serine, and then aspartic acid) and a helical pattern that is a defining feature of the BH3 motifs in the BCL-2 family proteins (Table 1) [20,21]. As proline is the fourth smallest amino acid by molecular weight, it was deemed a small amino acid in the case of the putative BH3 domain in p53 [22]. Given that the distance between leucine (L) and aspartic acid (D) in the BH3 domain is critical for its function, the predicted distance between these residues was analyzed to assess whether it falls within a functional range. Thus, the structures of the predicted canonical BH3 domains from five BCL-2 family proteins and the TAD from p53 were predicted using ESMFold, and the lengths between L and D of each domain were predicted using ICN3D’s Distance feature (Figure 3). As the distance between WT p53’s leucine and aspartic acid residues fell within the canonical BH3 domain range (8.7–15.0 Å), it was concluded that the p53 TAD has the potential to act as a BH3 domain, given its match of the BH3 motif [20,21]. This set of predicted data, along with the data from previous studies focusing on p53 TAD and its interactions with the BCL-2 family members [7], supports the conclusions of prior studies that p53 TAD may play an important role in directly interacting with and regulating the function of pro-death and pro-survival BCL-2 family proteins by acting as a BH3 domain.

### 2.3. Prediction and Visualization of Wild-Type and Mutant p53 Monomers Interacting with BCL-2 Yields Similar Outcomes from In Vitro Experiments

We next decided to visualize the protein–protein interactions of p53 with the BCL-2 family members. First, the ability of ESMFold to produce confident protein structures from a UNIPROT amino acid sequence [23] or a modified version of that UNIPROT sequence was validated (Figure 2A and Table S2). While ESMFold generated structures with some lower-confidence regions, this reflects the disordered nature of those areas. The disordered regions in the p53 TAD were verified by analyzing its amino acid sequence through ESMFold. Additionally, AlphaFold was used as an alternative protein-structure predictor for comparison (Figures S1 and S2 and Table S2).

**Figure 4:**
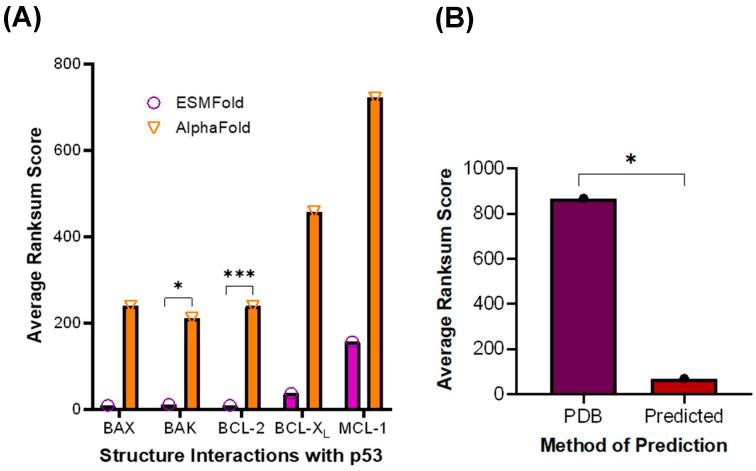
Structures predicted by ESMFold are statistically more confident than those predicted by AlphaFold. (**A**) Average Ranksum Scores for structure interactions comparing p53 and the BCL-2 family proteins with AlphaFold and ESMFold. (**B**) Average Ranksum Scores for PDB structure predicted interactions (PDB) and Predicted Structures’ predicted interactions (Predicted). * *p* < 0.05, *** *p* < 0.01.

To achieve a clearer picture of what interactions may occur in the absence of stabilizing mutations, the structures of p53 and the BCL-2 family proteins generated by AlphaFold or ESMFold were analyzed using the LZERD web server (West Lafayette, IN, USA) to predict interactions with WT p53, as well as p53 R273C and p53 R273H, which are common p53 variants in cancer patients [24]. Once the service returned a list of potential complexes, the complex with the lowest Ranksum score was selected, indicating the highest overall confidence [25,26,27,28,29]. The Ranksum score was also used to identify whether AlphaFold or ESMFold generated more statistically confident interaction predictions. There was a significant difference in average Ranksum scores between AlphaFold and ESMFold for predicting interactions between WT p53 and BCL-2 or BAK (Figure 4A), suggesting that ESMFold produces more reliable predictions, as its Ranksum score averages were lower. While BAX had similar averages to those of BCL-2 and BAK, there was a significant variation in its Ranksum scores, thus BAX was not selected for continued study. Given the low variation in Ranksum scores for the p53 and BCL-2 interaction, this complex was chosen for further pursuit. For the other BCL-2 family proteins, the Ranksum scores were much higher and thus rendered a *p*-value for the comparison above 0.05 (Figure 4A). This process was repeated with structures from the PDB, which produced significantly higher Ranksum scores than the predicted structures, supporting the use of the predicted structures rather than the PDB structures (Figure 4B).

**Figure 5:**
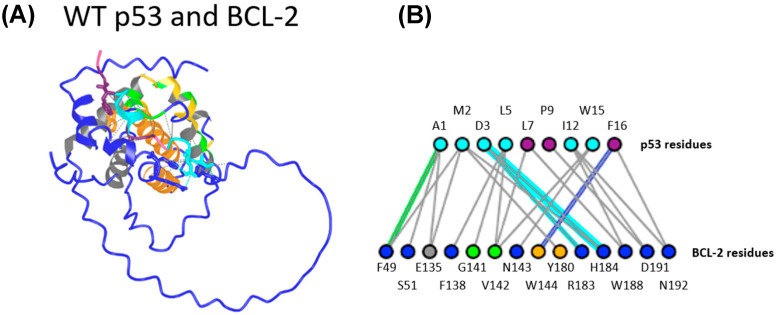
A predicted interaction between WT p53 (amino acids 39-57) and BCL-2 peptides derived from Ha et al. [7]. (**A**) WT p53 and BCL-2 peptide interaction prediction using our model. (**B**) WT p53 residues are the top row (amino acid 1 of the peptide corresponds to amino acid 39 of full-length p53), and BCL-2 residues are the bottom row. All residues not colored dark blue are residues identified by Ha et al. For p53 (**top row**), cyan residues are TAD2 residues. Purple residues are active p53 residues. For BCL-2 (**bottom row**), green residues are active residues, gray residues are directly involved in hydrophobic helices, and yellow residues are passive residues.

The newly predicted heterodimers of WT p53/BCL-2 peptides were analyzed using the Interactions tool in the ICN3D server to identify potential amino acid pairs between the peptides. The results were then cross-checked with previous studies that focused only on TAD interactions with BCL-2 [6,7,30,31,32], and with predictions using p53 TAD and BCL-2 amino acid sequences from Ha et al. [7] (Figure 5A). According to Ha et al., p53 amino acids 7, 8, 9, 13, 16, and 17 (corresponding to amino acids 46, 47, 48, 52, 55, and 56 in full-length p53) are active residues that are directly involved in protein–protein interactions. The BCL-2 amino acids 104, 109, 114, 129, 132, 141, 142, and 145 are also predicted as active amino acid residues [7]. Our results in Figure 5B were similar, but not identical, to the in vitro results seen in the literature [7]. The current model successfully identified the active residues 7, 9, and 16 in Ha et al.’s p53 peptide. For the BCL-2 peptide, the model predicted interactions with active residues G141 and V142 (Figure 5B).

**Figure 6:**
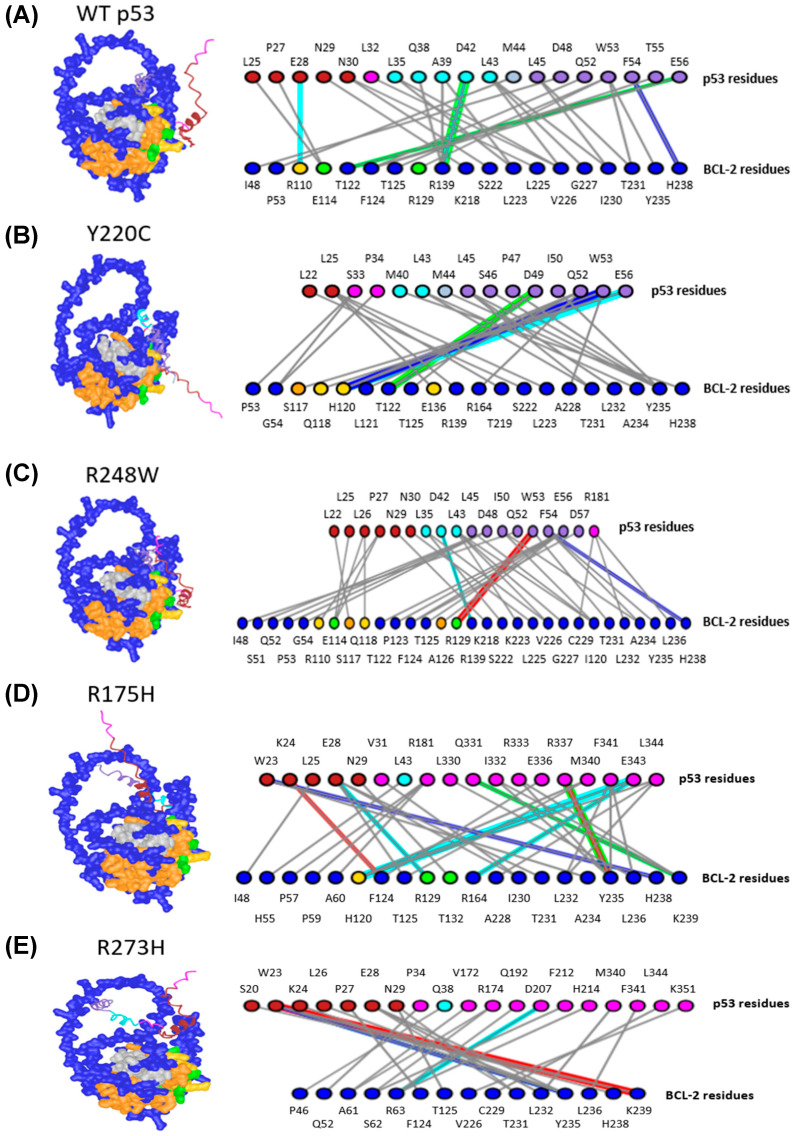

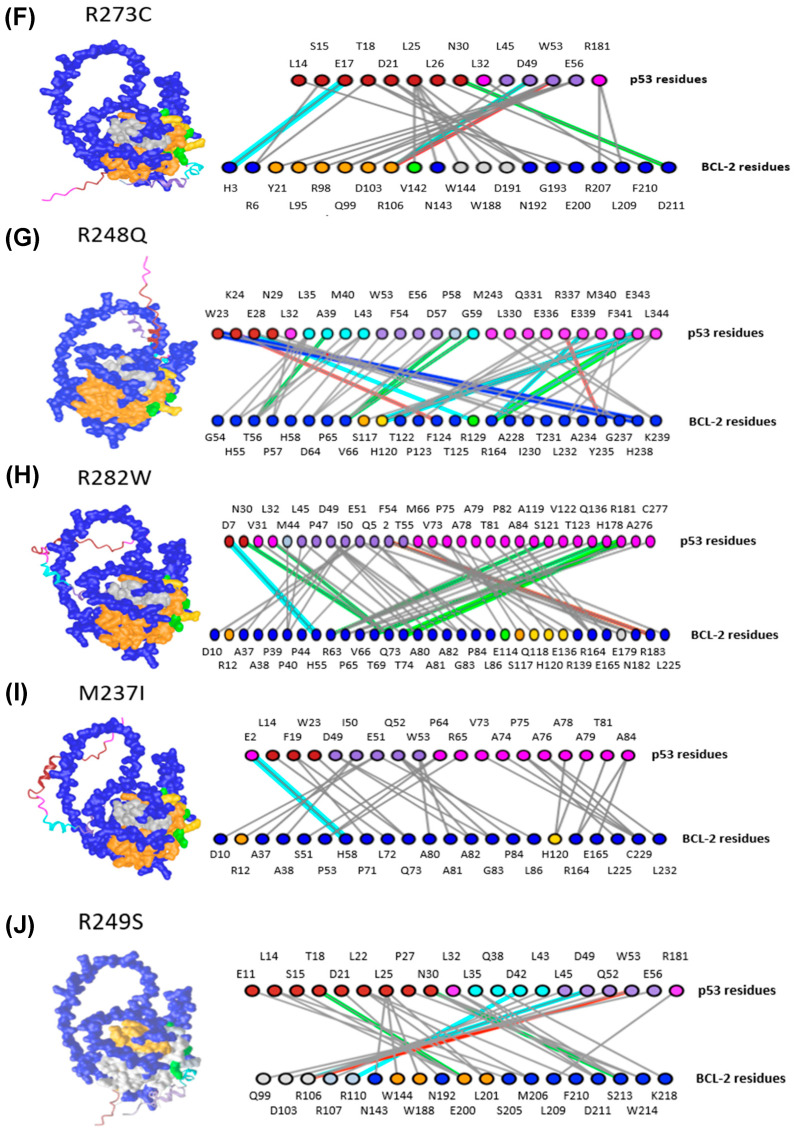
p53 Mutations Alter Interactions with BCL-2. (**A**) WT p53, (**B**) p53 Y220C, (**C**) p53 R248W, (**D**) p53 R175H, (**E**) p53 R273H, (**F**) p53 R273C, (**G**) p53 R248Q, (**H**) p53 R282W, (**I**) p53 M237I, (**J**) p53 R249S. All residues not colored pink or blue are residues identified by Ha et al. [7] For p53 (**top row**), dark red and cyan residues belong to TAD1 and TAD2, respectively. Purple and gray p53 residues are active and passive residues, respectively. For BCL-2 (**bottom row**), green and yellow residues are active and passive residues, respectively. Blue-gray residues are directly involved in hydrophobic helices, and orange residues are in helices involved in creating the hydrophobic pocket. The colors of lines are designated as follows: Green: H-bonds, Cyan: salt bridges or ionic bonds, Gray: contacts, Red: pi-cation interactions, Blue: pi-stacking interactions.

For the prediction of the full-length WT p53 monomer and BCL-2 interaction, E114 and R129 in BCL-2 were identified as active residues and R110 as a passive residue. Active and passive residues are defined as molecules directly and indirectly, respectively, involved in a protein–protein interaction with p53 (Figure 6A). Ha et al. identified that the BCL-2 amino acids 104, 109, 114, 129, 132, 141, 142, and 145 were all active residues in its interaction with a p53 TAD peptide, indicating that 2 out of 8 active residues were noted in our model. While the p53 amino acids 45, 46, 47, 48, 49, 50, 51, 52, 53, 54, 55, 56, and 57 were active residues in Ha et al. [7], our prediction was more successful at identifying residues in p53 that could interact with BCL-2; 7 out of 13 (L45, D48, Q52, W53, F54, T55, and E56) active p53 residues were noted in the WT p53 prediction (Figure 6A). These results indicate that while there is some variation between our prediction and what has been seen in vitro, this method can identify critical regions for binding and, to an extent, identify individual amino acids that could influence the interaction of the monomers.

Since these results identified the same regions and the majority of the key WT p53 amino acids listed in crystallography experiments [7], the analysis was extended to investigate the effects of MT p53 on BCL-2 interactions. Upon comparison of the WT p53-BCL-2 and the MT p53-BCL-2 predictions, a range of changes in the interaction sites were found, including an increased number of DBD amino acids between BCL-2 and p53 DBD variants R248W, R248Q, and R282W (Figure 6C,G,H). We also observed changes in the regions of BCL-2 that MT p53 was predicted to interact with, compared to WT p53. Y220C, R273C, R282W and R249S were predicted to interact with a higher number of passive residues of BCL-2 (Figure 6B,F,H,J). Other amino acids in hydrophobic helices were found to interact with p53 variants R248W (predicted to interact with BCL-2 amino acids S117 and A126), R273C (predicted to interact with BCL-2 amino acids Y21, L95, R98, Q99, D103, and R106), R282W (predicted to interact with BCL-2 amino acids R12 and S117), and R249S (predicted to interact with BCL-2 amino acids W144, W188, E200, and L201) (Figure 6C,F,H,J). Finally, variants R248W, R175H, R273H, R273C, and M237I were identified to have amino acids from other p53 regions interacting with BCL-2, including the proline-rich region, the DBD, and the C terminus (Figure 6C–F,I). The change in the predicted location and number of interactions between BCL-2 and p53 variants indicates that key domains involved in p53 functionality may become otherwise occupied by BCL-2 interactions, potentially further impairing p53’s ability to influence cellular processes. Compared to the data gathered with the WT p53 monomer and BCL-2, this data indicates that single amino acid variations in p53 are potentially sufficient to alter both the number and location of BCL-2-p53 interactions through conformational changes in p53 structure. Overall, these results support the hypothesis that p53 TADs may play an important role in mediating direct interactions with BCL-2 that can change upon p53 mutation, which may result in changes regulating cancer cell survival. Furthermore, since the predicted structures produced reasonably confident complexes that involved many of the amino acids discussed in prior literature, this methodology provides confident results based on structural predictions from protein folding algorithms.

### 2.4. Affinity Prediction of p53 and BCL-2 Demonstrates Relatively Strong Affinity Between Proteins

**Figure 7:**
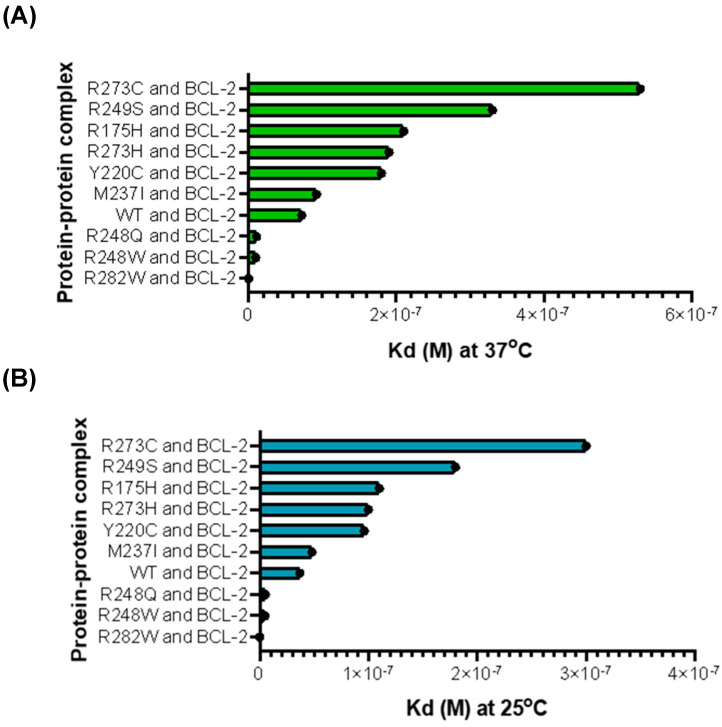
Predicted effects of p53 mutation on affinity with BCL-2 at 37 °C and 25 °C. (**A**) p53 and BCL-2 PRODIGY predicted affinity at 37 °C. (**B**) p53 and BCL-2 PRODIGY predicted affinity at 25 °C.

To further elucidate the potential effects of p53 on its interactions with BCL-2, a web server called PRODIGY was used (Utrecht, The Netherlands), which predicts affinity between molecules in a complex without being overly sensitive to interaction-induced conformational changes [31]. Overall, the relative ranking of variant affinity stayed in the same order when the temperature changed (Figure 7). However, the predicted affinities between p53 variants and BCL-2 were clearly stronger at 25 °C than 37 °C (95% Kd CI for 37 °C [1.5025 × 10^−7^, 3.6799 × 10^−7^], 95% Kd CI for 25 °C [2.0607 × 10^−7^, 8.5966 × 10^−8^, 95% CI for ΔG, [−11.259053, −9.2136745]) (Table S3). Despite the order remaining the same, there was a general trend towards lower affinities between the MT p53 monomer and BCL-2 monomer compared with the WT p53 monomer when predicted at 37 °C. This indicates that at physiological temperature, there is generally a decreased chance of MT p53 monomers interacting with BCL-2 monomers. These results also indicate that temperature impacts the affinity between all p53 variants and BCL-2. To predict whether these structures remained viable in solution, we used the GROMACS software version 2025.3 (Stockholm, Sweden) to analyze the stability of the protein–protein interactions between each p53 variant and BCL-2 for 2 nanoseconds (Figure S3) [33,34,35,36]. After analyzing the radius of gyration (Rg) and the Root Mean Square Deviation (RMSD) for each complex, we concluded that the predicted complexes remained compact and stable across the 2-nanosecond time frame, indicating that the complexes of p53 variants and BCL-2 are stable in solution.

## 3. Discussion

Using pre-established computational methods, we were able to reasonably estimate the interactions and affinities between ESMFold-generated p53 monomer variants and BCL-2. Given that this method returns the results similar to those seen in the previous in vitro experiments, the new method in silico can be used as a tool to assist in predicting the outcome of wet lab experiments focusing on protein–protein interactions [7]. There is in vitro evidence that supports p53 TAD binding to the BH3 groove of the BCL-2-family proteins and the idea that p53 may play a role in controlling pro-survival activity of BCL-2 by direct interactions of its TAD or TAD analogs with BCL-2’s BH3-binding pocket [6,7,30,31,32]. Major variants of p53 are predicted to have a decrease in their affinity for direct interactions with BCL-2 (Figure 7), pointing to a potential increase in BCL-2 activity and thus an increase in cancer cell survival. This decrease in affinity is likely due to the alterations in the structure of the p53 monomer caused by mutations in p53 DBD; this theory is supported by the variants with physically similar amino acids having similar affinities to WT p53 with BCL-2 and those with physically different amino acids having much weaker affinities compared to WT p53. However, the GROMACS simulation data indicate that the complexes are predicted to be stable in solution despite the variances in affinity, suggesting a physiological relevance.

While these results are of interest, it is important to note that only a small fraction (29%) of p53 remains in monomer form before any DNA damage occurs [37]. When the cell undergoes DNA damage, the vast majority of p53 oligomerizes to a tetrameric form [37]. This change in the p53 oligomerization state depending on DNA damage suggests that there remains significant work to be done on predicting the ability of a p53 dimer or tetramer to interact with BCL-2 and other BCL-2 family members, which may be more likely to occur. Thus, studying the interactions between BCL-2 and the p53 dimer or tetramer is one possible avenue for future studies. A second direction would be investigating the relation between the sensitivity/resistance of chemotherapy/targeted therapy and the interaction of p53 and the BCL-2 family proteins. We and others have demonstrated that MT p53 expression is associated with chemo- and radio-resistance in a variety of cancers [38,39,40,41]. A third direction would be exploring the effects of the p53-BCL-2 interaction on the tumor microenvironment, specifically vascularization. A study by Perrone et al. notes a positive correlation between vascular endothelial growth factor (VEGF) [42], p53, and BCL-2, and another study notes BCL-2’s importance in the progression of angiogenesis [43]. This leads to a question about the role of the p53-BCL-2 interaction in tumor vascularization. A fourth direction to eventually explore would be studying Li-Fraumeni p53 variants. While we examined p53 variants such as R175H, R248Q, R248W, R273H, and R282W that are noted as being involved in Li-Fraumeni syndrome, other mutations, such as H179Y, R213Q, and G245S and how they interact with BCL-2, remain to be studied [44].

In conclusion, this work shows that the procedure demonstrated here can generate reliable results that are similar to those observed in in vitro experiments. Thus, this method can potentially be used as a predictive workflow to inform future in vitro studies. This model also shows evidence of the TAD of monomeric WT p53 interacting with BCL-2 in a manner similar to that of a BH3-only BCL-2 family protein. Furthermore, these results also indicate that while there is some variation between this work and results seen in vitro, this method can identify critical regions, and to a lesser extent, specific amino acids involved in protein–protein interactions. Finally, the data also point to a reliance on steric similarities of MT p53 in the DBD to alter the affinity between the two proteins.

## 4. Materials and Methods

### 4.1. Protein Structure Prediction Using AlphaFold and ESMFold

The protein amino acid sequences were obtained from UNIPROT for p53, BCL-2, BCL-XL, MCL-1, BAX, and BAK (Table S2). Then, the sequences were put into the ESMFold option in ICN3D (NIH v2023_02) and the algorithm was run using the Google ColabFold code for AlphaFold, and the same sequences were used for the ESMFold predictions. For each p53 mutant, the wild-type sequence from UNIPROT was obtained and the corresponding amino acid was changed to the mutant of interest. Then, the ESMFold and AlphaFold structure predictions were run. Finally, each structure was downloaded and uploaded to ICN3D, where they were independently visualized. The pLDDT score, which measures the per-residue local confidence through a local difference Cα test, was used to determine the overall reliability of the predicted structures [13]. Results were saved as .pdb files for the prediction step. pLDDT distributions were generated using GraphPad Prism version 10.

Google ColabFold and ESMFold parameters remained unchanged from the original code. For ColabFold, the settings were as follows: 3 recycles, unchanged MSA settings, and relaxation was used without the GPU. MSA options were left at original settings, and the random seeds for each top result were 0.

### 4.2. FATCAT Superposition

The predicted WT p53 and BCL-2 structures were put into the HOMOLOGY SEARCH function on the web server FATCAT [13,14,15,16,17]. After running the search function, the top PDB structure was identified as our prediction based on the server’s sorting of best matches. This tool returned the most similar experimentally determined Protein Data Bank (PDB) structures in a table ranked from most similar to least similar based on *p*-value (Table S1).

### 4.3. Protein–Protein Interaction Prediction

Using the LZerD server [26], one p53 monomer and one BCL-2 family member were input before running the prediction. This server scored in the top 10 of the CAPRI protein assembly prediction challenge, making it an attractive server for the workflow [45]. The Ranksum score is a sum of three other scoring mechanisms (GOAP, DFIRE, and ITScore), which allows for a more comprehensive ranking of each predicted complex [28]. Once completed, the result with the lowest Ranksum score was downloaded and visualized in ICN3D. Using ICN3D’s interactions function under the analysis tab, a map of amino acid interactions between p53 and BCL-2 family members was obtained. These results were compared to those found in in vitro studies focusing on the TAD of p53 and BCL-2 family members to ensure models identified multiple amino acids involved in the interactions that were discussed in the in vitro studies.

### 4.4. Identification of Optimal Structure Prediction Method

After obtaining the ESMFold and AlphaFold predictions of the BCL-2 family proteins interacting with WT p53, p53 R273C, and p53 R273H, the three p53 variants’ Ranksum scores for each BCL-2 family member were averaged and graphed using GraphPad Prism version 10. Then, the ESMFold average Ranksum score was compared to the AlphaFold Ranksum score using a *t*-test, with *p* < 0.05.

### 4.5. Protein–Protein Interaction Affinity Prediction

Using the web server https://bianca.science.uu.nl/prodigy/ (accessed on 21 December 2025) [31], the LZerD interaction prediction was uploaded to the server, identifying p53 as chain A each time. Then, the service at either 37 °C (the average human body temperature) or 25 °C (room temperature) was run and the results were analyzed using GraphPad Prism version 10. All other experimental conditions, such as pH, ionic strength, and pressure, remained as constant as set by the PRODIGY server [31]. Error bars are the standard error.

### 4.6. Molecular Dynamics Simulations

Following the procedure as outlined in the Lysozyme in Water tutorial by Lemkul [46], a topology of each p53 variant/BCL-2 complex was created using the Terminal command line. Then, a solvent was introduced to the created cubic box and ions were added to make the solution neutral. The energy was minimized to a maximum force of 1000 kj/mol/nm with 50,000 steps. Parameters for this minimization can be found in the minim.mdp file. Once completed, the simulation was equilibrated using canonical NVT and NPT ensembles. The parameters can be found in the nvt.mdp and npt.mdp files. Finally, the simulation for 2 nanoseconds (ns) was run. Parameters for the 2 ns run can be found in the md2ns.mdp file. The simulation was repeated twice per p53 variant/BCL-2 complex and analyzed the radius of gyration (Rg) and the root mean square deviation (RMSD) using GraphPad. All files can be found in. Figshare, identifier https://doi.org/10.6084/m9.figshare.30270007.

## Figures and Tables

**Table 1:** Amino acids in the BH3 domain of the BCL-2 family proteins and a putative BH3 motif in p53 TAD. Cyan highlights are amino acids with similar chemical characteristics to other amino acids in their same aligned position. Red highlights are the leucine and aspartic acid residues that play a key role in the BH3 domain structure and function.

Protein	Amino Acid Range	Sequence
BCL-2	93–106	**VHLTLRQAGDDFSR**
BAD	110–123	**YGRELRRMSDEFVD**
BCL2L14	212–225	**IVELLKYSGDQLER**
NDV	88–101	**LTTLLTPLGDSIRR**
BCL-XL	86–99	**VKQALREAGDEFEL**
NOXA	25–38	**CATQLRRFGDKLNF**
BIM	148–161	**IAQELRRIGDEFNA**
p53	39–52	**AMDDLMLSPDDIEQ**

## Data Availability

Protein sequences can be found on UNIPROT. The ESMFOLD algorithm can be located on ICN3D.com. The interaction prediction algorithm can be found on https://lzerd.kiharalab.org/upload/upload/ (accessed on 1 September 2025). PRODIGY prediction can be found on https://rascar.science.uu.nl/prodigy/ (accessed on 1 September 2024). FATCAT HOMOLOGY alignment service is located at https://fatcat.godziklab.org/ (accessed on 1 August 2025). Raw data, protein–protein interaction PDB files and code files can be found in Figshare, identifier https://doi.org/10.6084/m9.figshare.30270007.

## Supplementary Materials

The following supporting information can be downloaded at: https://www.mdpi.com/article/10.3390/ijms27010244/s1.
