# Supplementary material for: A Computational Approach for the Prediction of p53 and BCL-2 Protein–Protein Interactions
Source: Int J Mol Sci. 2025 Dec 25;27(1):244. doi: 10.3390/ijms27010244 (PMC12785448; doi:10.3390/ijms27010244)
Supplement: Supplementary file 1 [file ijms-27-00244-s001.zip › ijms-3937497-supplementary.pdf]

Supplementary Materials:

Figure S1:

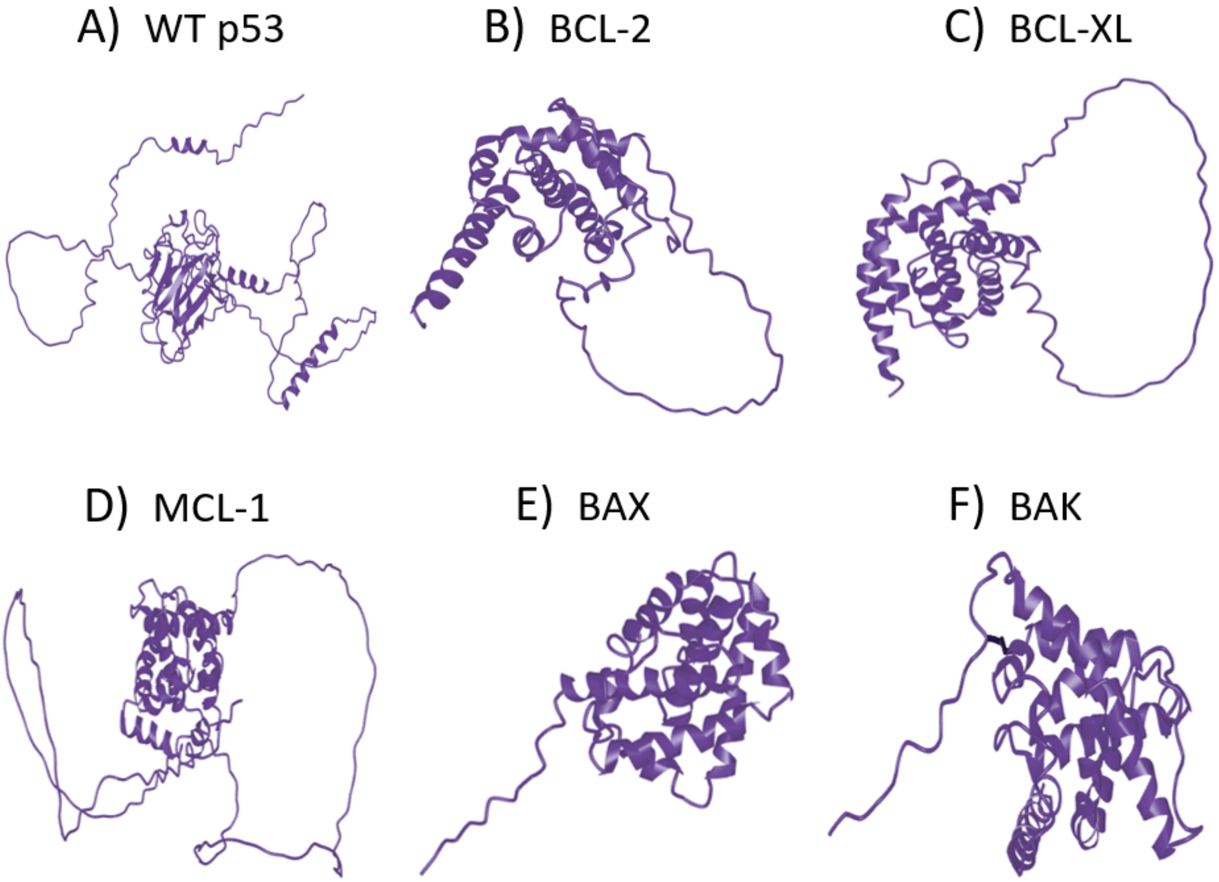

Figure S1: AlphaFold Structures of p53 and BCL-2 family member proteins.
A) WT p53, **B)** BCL-2, **C)** BCL-XL, **D)** MCL-1, **E)** BAX, **F)** BAK

Figure S2:

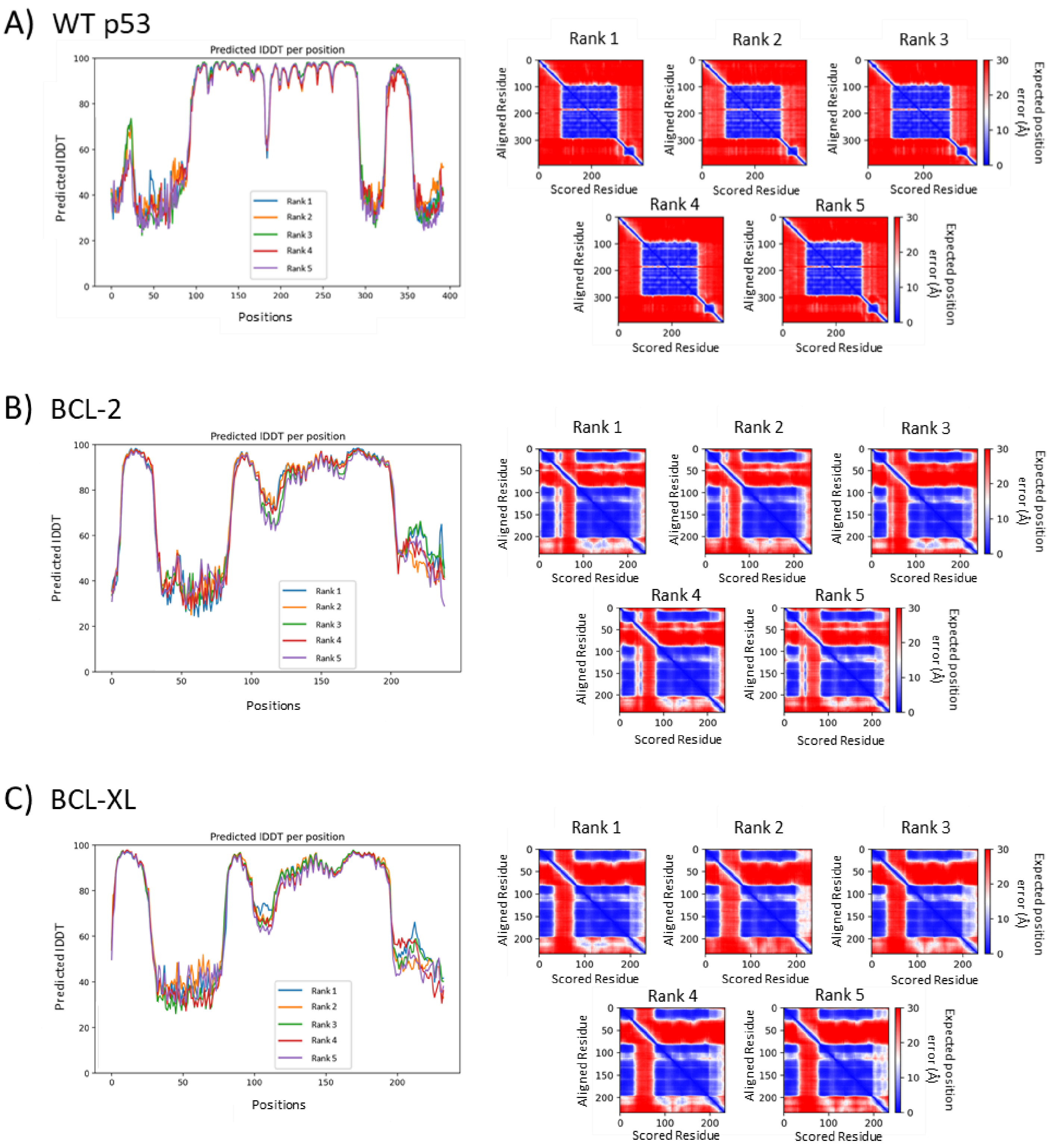
A) WT p53
Predicted lDDT per position
Predicted lDDT
Positions
Rank 1
Rank 2
Rank 3
Rank 4
Rank 5
Aligned Residue
Scored Residue
Expected position error (Å)
B) BCL-2
Predicted lDDT per position
Predicted lDDT
Positions
Rank 1
Rank 2
Rank 3
Rank 4
Rank 5
Aligned Residue
Scored Residue
Expected position error (Å)
C) BCL-XL
Predicted lDDT per position
Predicted lDDT
Positions
Rank 1
Rank 2
Rank 3
Rank 4
Rank 5
Aligned Residue
Scored Residue
Expected position error (Å)

D) MCL-1

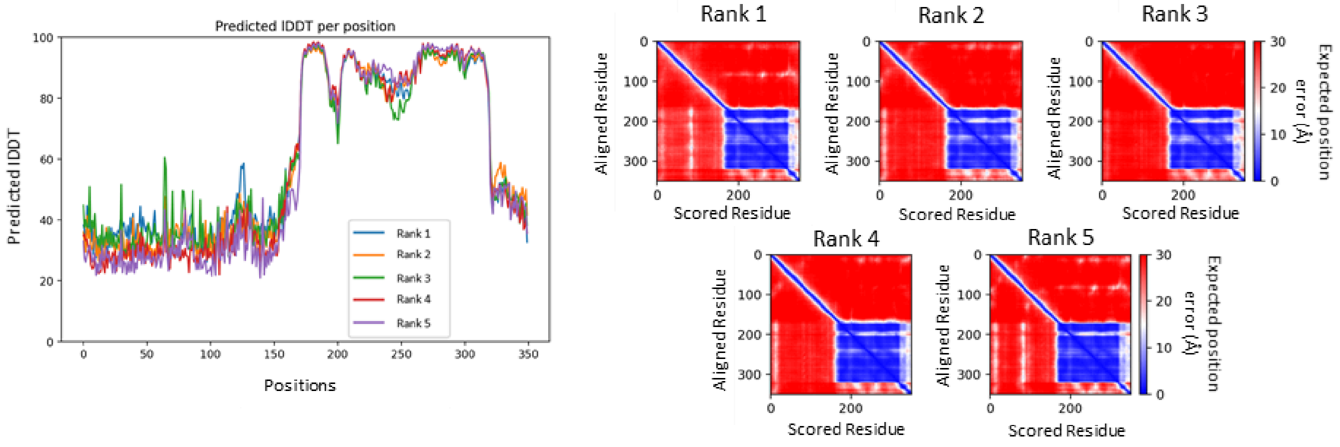

E) BAX

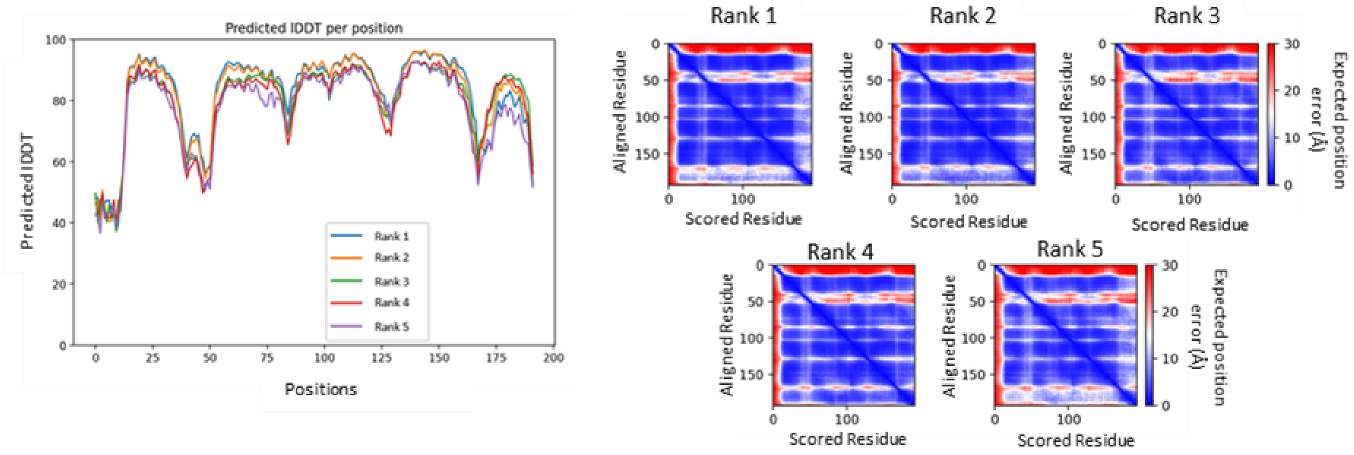

F) BAK

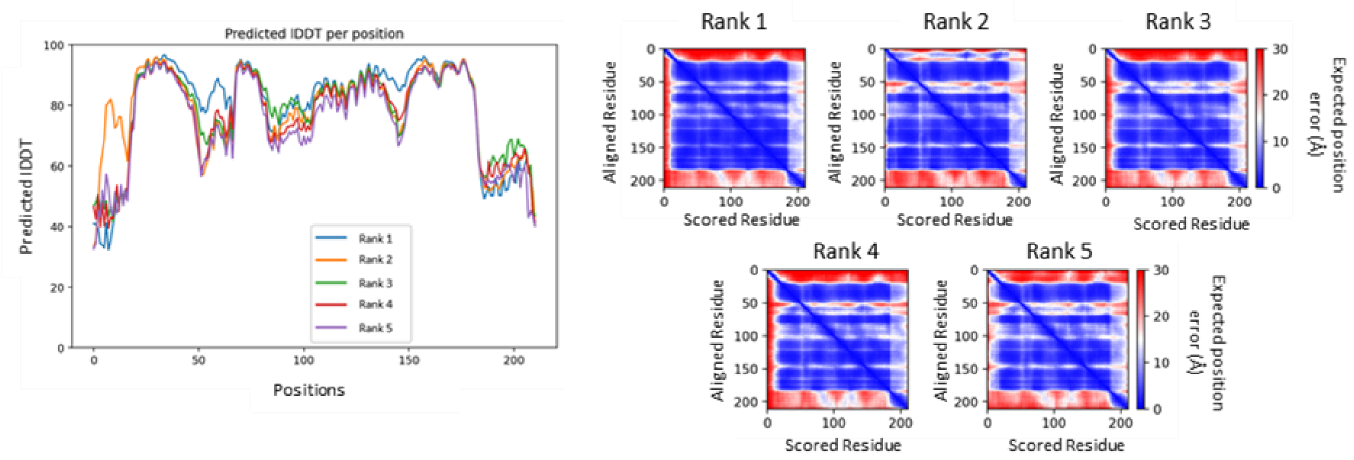

Figure S2: pLDDT and PAE graphs of AlphaFold Structures Demonstrate Confident Regions.
A) WT p53, **B)** BCL-2, **C)** BCL-XL, **D)** MCL-1, **E)** BAX, **F)** BAK

Figure S3:

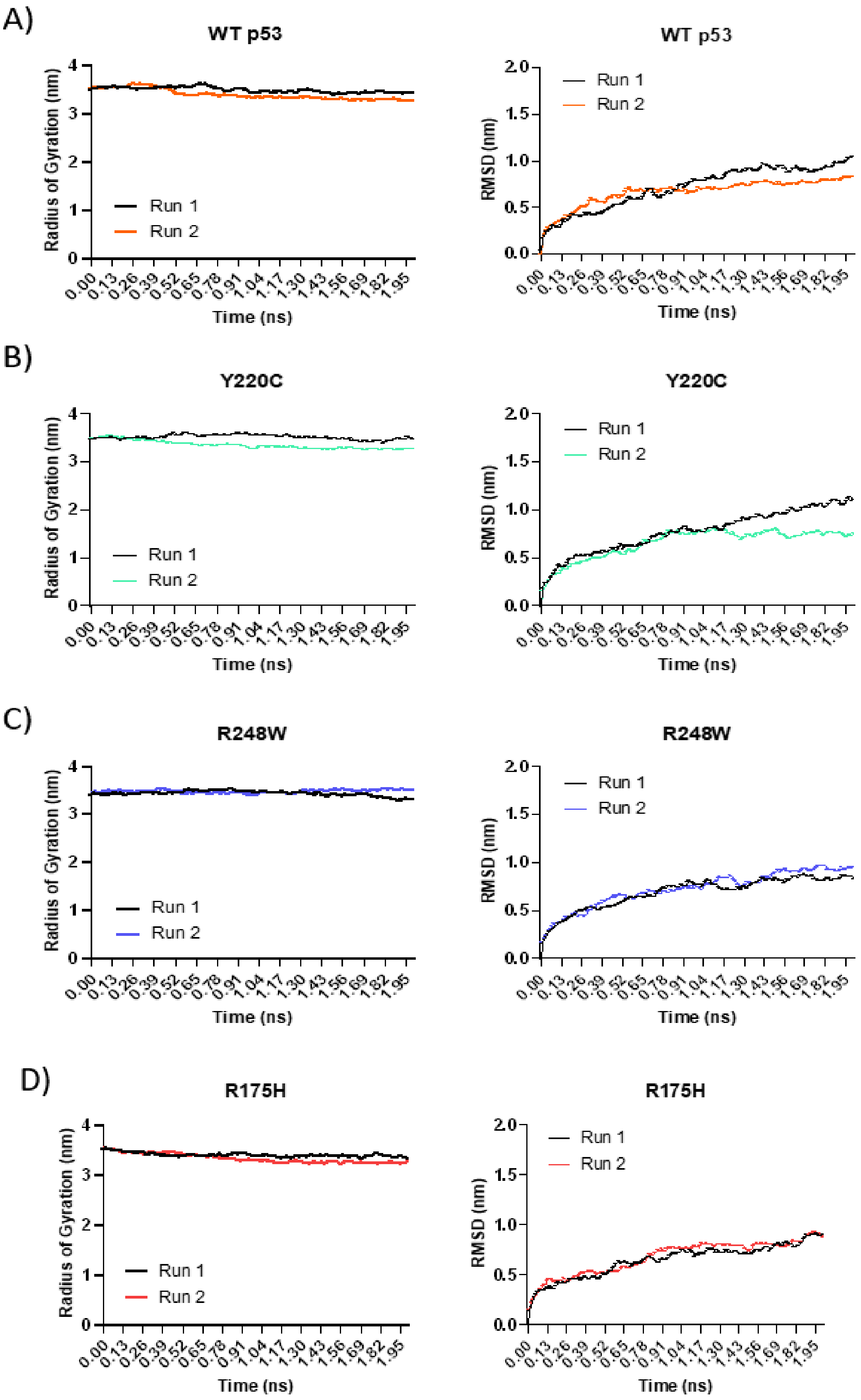
A)
WT p53
Radius of Gyration (nm)
Run 1
Run 2
Time (ns)
WT p53
RMSD (nm)
Run 1
Run 2
Time (ns)
B)
Y220C
Radius of Gyration (nm)
Run 1
Run 2
Time (ns)
Y220C
RMSD (nm)
Run 1
Run 2
Time (ns)
C)
R248W
Radius of Gyration (nm)
Run 1
Run 2
Time (ns)
R248W
RMSD (nm)
Run 1
Run 2
Time (ns)
D)
R175H
Radius of Gyration (nm)
Run 1
Run 2
Time (ns)
R175H
RMSD (nm)
Run 1
Run 2
Time (ns)

E)

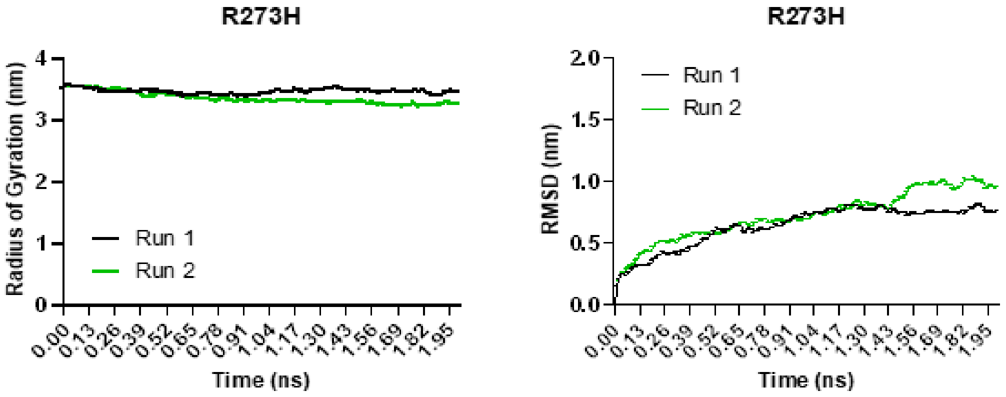
R273H
Radius of Gyration (nm)
Run 1
Run 2
Time (ns)
R273H
RMSD (nm)
Run 1
Run 2
Time (ns)

F)

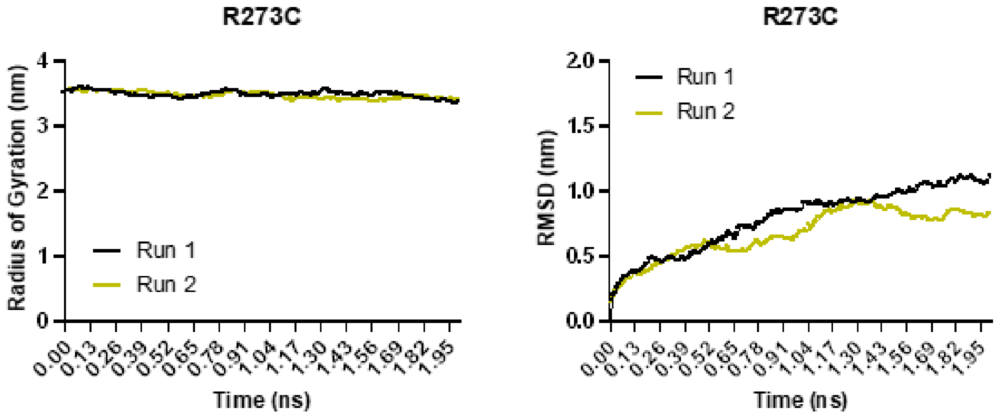
R273C
Radius of Gyration (nm)
Run 1
Run 2
Time (ns)
R273C
RMSD (nm)
Run 1
Run 2
Time (ns)

G)

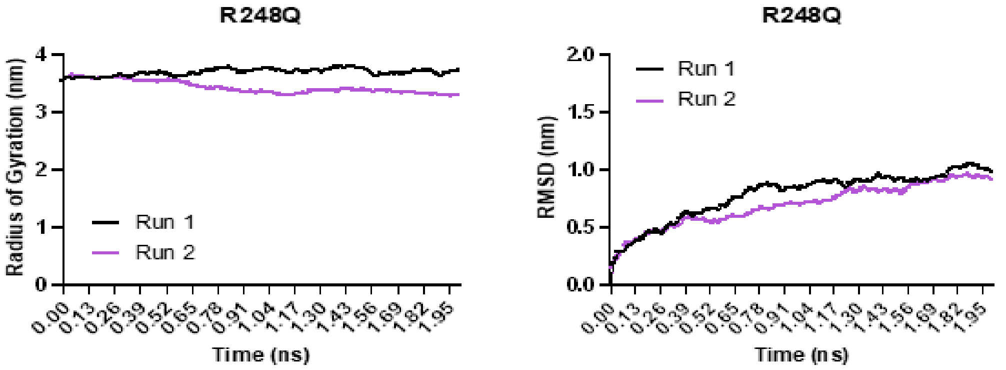
R248Q
Radius of Gyration (nm)
Run 1
Run 2
Time (ns)
R248Q
RMSD (nm)
Run 1
Run 2
Time (ns)

H)

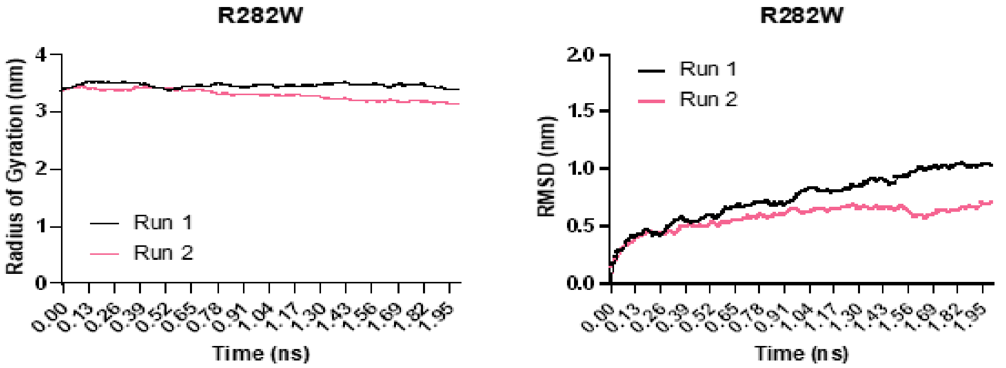
R282W
Radius of Gyration (nm)
Run 1
Run 2
Time (ns)
R282W
RMSD (nm)
Run 1
Run 2
Time (ns)

I)

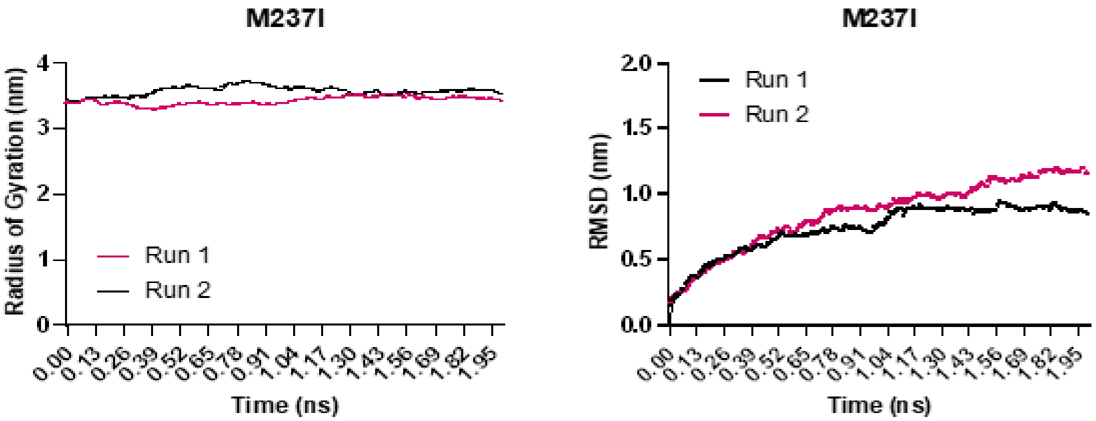

J)

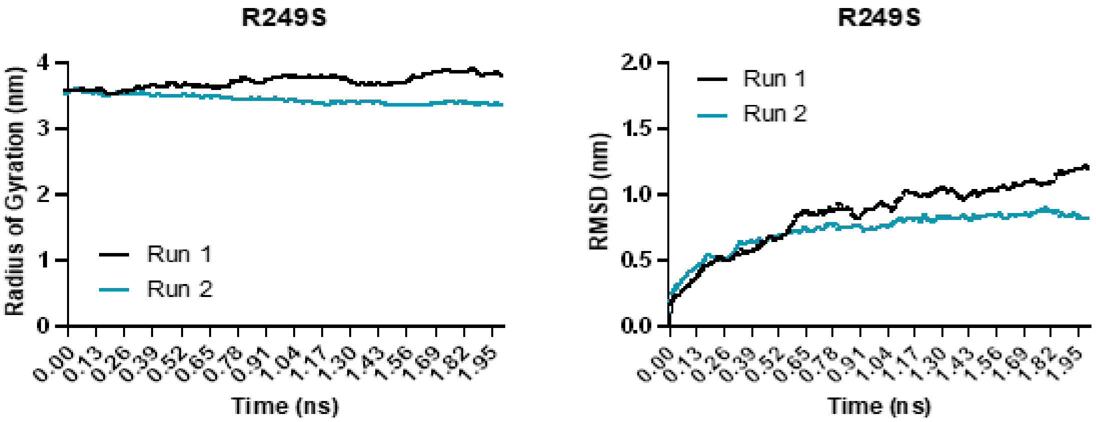

Figure S3: Radius of Gyration (Rg) and Root Mean Square Deviation (RMSD) graphs show p53 Variant Stability.
A) WT p53, **B)** Y220C, **C)** R248W, **D)** R175H, **E)** R273H, **F)** R273C, **G)** R248Q, **H)** R282W, **I)** M237I, **J)** R249S

Table S1: FATCAT alignment scores for WT p53 and BCL-2

A) WT p53, **B)** BCL-2

1: PDB identifier. 2: length of PDB structure. 3: p-value. 4: Number of gaps allowing for flexibility of protein structures. 5: length of sequence analyzed. 6: root mean square deviation of all alpha carbon atoms. 7: length of gaps. 8: Percent identity match. 9: Name of protein identified [13-17].

A) WT p53

1	2	3	4	5	6	7	8	9
**structure**	**length**	**P-value**	**twist**	**opt-len**	**opt-rmsd**	**gap**	**seq-ide(%)**	**UniProtKB-ID**
8r1fC	202	0	0	198	1.34	0	100	P53_HUMAN

B) BCL-2

structure	length	P-value	twist	opt-len	opt-rmsd	gap	seq-ide(%)	UniProtKB-ID
5jsnC	149	0.00E+00	0	148	1.4	54	72.77	BCL2_HUMAN
5jsnA	149	0.00E+00	0	148	1.35	54	72.77	BCL2_HUMAN
2w3lB	142	0.00E+00	0	142	0.93	54	68.88	BCL2_HUMAN
2w3lA	141	0.00E+00	0	141	1.12	57	68.69	BCL2_HUMAN
6qg8A	137	0.00E+00	0	137	0.72	57	62.89	BCL2L1_HUMAN
7yb7A	136	1.11E-16	0	136	1.37	58	62.37	BCL2_HUMAN
7yb7B	138	2.22E-16	0	138	1.38	57	62.56	BCL2_HUMAN
6iwbD	139	2.51E-14	0	139	1.47	58	69.54	BCL2_HUMAN
8u27A	177	3.95E-14	0	169	2.73	53	71.62	BCL2_HUMAN
6iwbB	139	5.73E-14	0	139	1.58	58	69.54	BCL2_HUMAN
8hlmB	139	7.90E-14	0	139	1.95	57	62.24	BCL2_HUMAN
5fcgA	150	7.40E-05	2	145	4.64	49	56.7	BCL2_HUMAN

Table S2: UNIPROT sequences and IDs used for predictions

Protein	UNIPROT ID	Sequence
WT p53	P04637	MEEPQSDPSVEPPLSQETFSDLWKLLPENNVLSPLPSQAMDDLMLSPDDIEQWFTEDPGPDEAPRMP EAAPPVAPAPAAPTPAAPAPAPSWPLSSSVPSQKTYQGSYGFRLGFLHSGTAKSVTCTYSPALNKMFC QLAKTCPVQLWVDSTPPPGTRVRAMAIYKQSQHMTEVVRRCPHHERCSDSDGLAPPQHLIRVEGNL RVEYLDDRNTFRHSVVVPYEPPEVGSDCTTIHYNYMCNSSCMGGMNRRPILTIITLEDSSGNLLGRNS FEVRVCACPGRDRRTEEENLRKKGEPHHELPPGSTKRALPNNTSSSPQPKKKPLDGEYFTLQIRGRER FEMFRELNEALELKDAQAGKEPGGSRAHSSHLKSKKGQSTSRHKKLMFKTEGPDSD
BCL-2	P10415	MAHAGRTGYDNREIVMKYIHYKLSQRGYEWDAGDVGAAPPGAAPAPGIFSSQPGHTPHPAASRDP VARTSPLQTPAAPGAAAGPALSPVPPVVHLTLRQAGDDFSRRYRRDFAEMSSQLHLTPFTARGRFATV VEELFRDGVNWGRIVAFFEFGGVMCVESVNREMSPLVDNIALWMTEYLNRHLHTWIQDNGGWDAF VELYGPSMRPLFDFSWLSLKTLLSLALVGACITLGAYLGHK
BCL-XL	Q07817	MSQSNRELVVDFLSYKLSQKGYSWSQFSDVEENRTEAPEGTESEMETPSAINGNPSWHLADSPAVNG ATGHSSSLDAREVIPMAAVKQALREAGDEFELRYRRAFSDLTSQLHITPGTAYQSFEQVVNELFRDGV WGRIVAFFSFGGALCVESVDKEMQVLVSRIAAWMATYLNDHLEPWIQENGGWDTFVELYGNNAAA ESRKGQERFNRWFLTGMTVAGVVLLGSLFSRK
BAX	Q07812	MDGSGEQPRGGGPTSSEQIMKTGALLLQGFIQDRAGRMGGEAPELALDPVPQDASTKKLSECLKRIG DELDSNMELQRMIAAVDTDSPREVFFRVAADMFSDGNFNWGRVVALFYFASKLVLKALCTKVPELIR TIMGWTLDFLRERLLGWIQDQGGWDGLLSYFGTPTWQTVTIFVAGVLTASLTIWKKMG
BAK	Q16611	MASGQGPGPPRQECGEPALPSASEEQVAQDTEEVFRSYVFYRHQQEQEAEGVAAPADPEMVTLPLQP SSTMGQVGRQLAIIGDDINRRYDSEFQTMLQHLQPTAENAYEYFTKIATSLFESGINWGRVVALLGFG YRLALHVYQHGLTGFLGQVTRFVVDFMLHHCIARWIAQRGGWVAALNLGNGPILNVLVVLGVVLL GQFVVRRFFKS
MCL-1	Q07820	MFGLKRNAVIGLNLYCGGAGLGAGSGGATRPGGRLLATEKEASARREIGGGEAGAVIGGSAGASPPS TLTPDSRRVARPPPIGAEVPDVTATPARLLFFAPTRRAAPLEEMEAPAADAIMSPEEELDGYEPEPLGK RPAVLPLLELVGESGNNTSTDGSLPSTPPPAEEEEDELYRQSLEIISRYLREQATGAKDTKPMGRSGATS RKALETLRRVGDGVQRNHETAFQGMLRKLDIKNEDDVKSLSRVMIHVFSDGVTWGRIVTLISFGAFV AKHLKTINQE5CIEPI.AE51IDYJ.YRTKRDWI.VKORGWDGEYEEfHYEPLEGGTRNVTIAFAGVAGVG AGIAYLTR

Table S3: Confidence intervals for PRODIGY-derived ΔG and Kd data

A) ΔG Confidence Interval

	**37℃**	**25℃**
**Number of Samples**	10	10
**ΔG (kcal mol-1) STDEV**	1.65001377	1.65001377
**ΔG (kcal mol-1) AVG**	-10.236364	-10.236364
**STDEV/sqrt(N)**	0.52178017	0.52178017
**Z score**	1.96	1.96
**Z *STDEV/sqrt(N)**	1.02268913	1.02268913
**Confidence Interval X+**	-9.2136745	-9.2136745
**Confidence Interval X-**	-11.259053	-11.259053

B) Kd Confidence Interval

	**37℃**	**25℃**
**Number of Samples**	10	10
**Kd (M) at ℃ STDEV**	2.4242E-07	1.387E-07
**Kd (M) at ℃ AVG**	2.1773E-07	1.201E-07
**STDEV/sqrt(N)**	7.6661E-08	4.386E-08
**Z score**	1.96	1.96
**Z *STDEV/sqrt(N)**	1.5025E-07	8.5966E-08
**Confidence Interval X+**	3.6799E-07	2.0607E-07
**Confidence Interval X-**	6.7478E-08	3.4136E-08
